# Development of Liposomes That Target Axon Terminals Encapsulating Berberine in Cultured Primary Neurons

**DOI:** 10.3390/pharmaceutics16010049

**Published:** 2023-12-28

**Authors:** Ikuma Hori, Hideyoshi Harashima, Yuma Yamada

**Affiliations:** 1 Faculty of Health Sciences, Hokkaido University, Kita-12, Nishi-5, Kita-ku, Sapporo 060-0812, Japan; hori.i4@hs.hokudai.ac.jp; 2 Faculty of Pharmaceutical Sciences, Hokkaido University, Kita-12, Nishi-6, Kita-ku, Sapporo 060-0812, Japan; harasima@pharm.hokudai.ac.jp

**Keywords:** liposome, axon terminals, mitochondrial dynamics, axon outgrowth

## Abstract

Most of the energy in neurons is produced in mitochondria. Mitochondria generate the ATP that is essential for neuronal growth, function, and regeneration. Mitochondrial axonal transport plays a crucial role in maintaining neuronal homeostasis and biological activity. Decreased mitochondrial axonal transport at axon terminals, where the metabolism of substances is likely to be delayed, may contribute to neurological dysfunction. Therefore, regulation of mitochondrial dynamics at axon terminals has attracted considerable interest as a strategy to modulate neuronal function. Nanoparticles may be useful in controlling local mitochondrial dynamics. Nevertheless, there are few reports on the influence of drug delivery that nanoparticles impart on the mitochondrial dynamics in neurons. This paper reports the results of a study using liposomes (LPs) to examine local drug delivery and pharmacological actions on neurons. We tested berberine (BBR), which is an activator of AMP-activated protein kinase (AMPK), to examine the utility of this drug as a cellular energy sensor. Axon terminals targeting LPs were prepared. The amount of axon terminals targeting LPs was increased compared with treatment using cationic LPs. Moreover, axon terminal-targeting LPs increased anterograde transport by about 40% compared with that of either naked BBR or cationic LPs and suppressed axonal retraction. Our findings suggest that local drug delivery to neurons is important for enhancing pharmacological activity in axon terminals.

## 1. Introduction

Neurons are composed of cell bodies, dendrites, and axons. The primary functions of neurons include the biosynthesis and degradation of proteins, RNA, and organelles in cell bodies. Therefore, metabolism tends to decrease at axon terminals far from the cell body. Axonal transport is the mechanism that maintains the metabolism of axon terminals. In particular, axonal transport of mitochondria consists of anterograde transport to axon terminals and retrograde transport to the cell body. The balance between these two forms of transport depends on the state of the intracellular energy [1]. Axonal transport mainly makes use of energy produced in mitochondria, so mitochondrial quality plays an important role in maintaining the metabolism of other factors [2]. Experiments using optogenetics and microfluidic devices have demonstrated the utility of modulating local mitochondrial axonal transport [3,4]. The process, however, requires a surgical procedure, and its effects on the human body are not well understood, which is why this process has yet to be applied clinically.

The activation of AMP-activated protein kinase (AMPK), a cellular energy sensor, contributes to the regulation of mitochondrial dynamics and is involved in axogenesis [5]. The energy deficit at the axon terminals for axonal outgrowth is supplemented by increasing the anterograde transport from mitochondria to the axon terminals through the activation of AMPK. Therefore, mitochondrial energy maintenance is critical for neuronal development, regeneration, and synaptic function [6,7]. Axotomy is known to cause a local decrease in mitochondrial motility accompanied by ATP depletion, which limits axonal regeneration [8]. Berberine (BBR), which exhibits neuroprotective effects, is one of the activators of AMPK [9]. BBR is known to affect neurites, which are important for the maintenance of nerve function [10,11,12]. However, the effects that local BBR delivery exert on mitochondrial dynamics and on neurites remains to be elucidated.

Local delivery of BBR to axon terminals using liposomes (LPs) could regulate mitochondrial dynamics. Previous studies have examined drug delivery using nanoparticles to target postsynaptic receptors [13,14]. In the present study, therefore, we evaluated whether modification of the nanoparticle surface could enable local drug delivery to presynaptic axon terminals. We used MITO-Porter cationic LPs developed in our laboratory [15,16]. We found that efficient delivery of BBR using MITO-Porter enhances neuroprotective effects [17]. We changed the surface modification of MITO-Porter and attempted to develop LPs for local drug delivery to axon terminals. Herein, we describe the intracellular localization of LPs modified with peptides targeting axon terminals, as well as the intracellular localization of MITO-Porter modified with octaarginine (R8) in the culture of primary neurons.

The purpose of this study was to prepare LPs that could enhance local delivery of BBR to axon terminals and to evaluate whether these LPs would affect mitochondrial dynamics and axon length in neurons. We hypothesized that cationic substances such as BBR would bind non-specifically to cell membranes because they are negatively charged. Therefore, we considered that modifying LPs with peptides targeting presynaptic sites would enable local drug delivery to axon terminals. The use of this system reveals the importance of local drug delivery in enhancing the pharmacological effect on neurons due to reduced nonspecific binding. In the present study, axon terminal-targeting LPs were prepared, and their physicochemical properties were evaluated.

**Figure 1:**
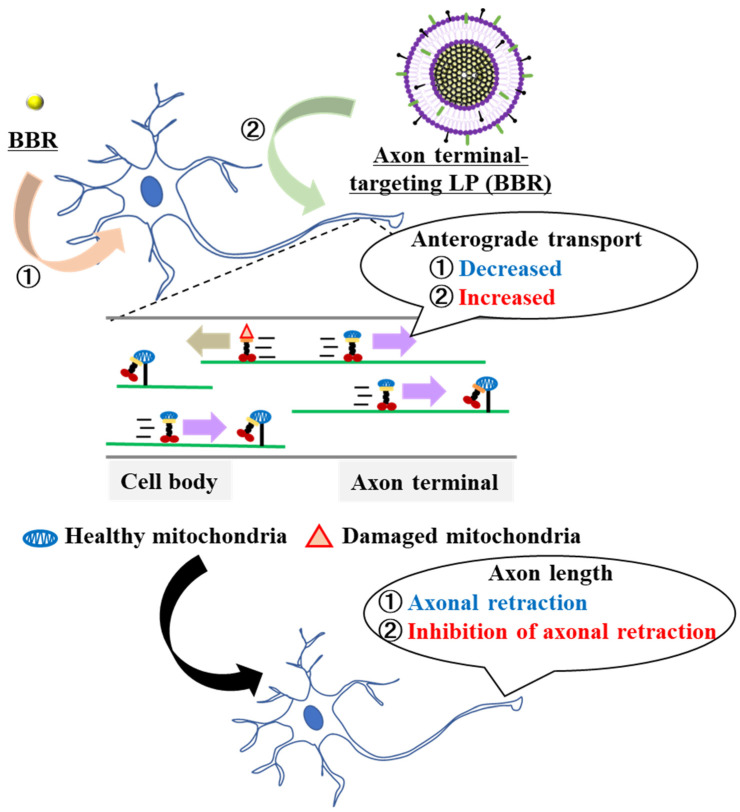
Schematic diagram showing the local delivery of BBR employing the liposome system. Accumulation of BBR in the cell body induced a decrease in the axonal mitochondria transport. Furthermore, it promoted axonal retraction. The use of axon terminal-targeting LPs (BBR) resulted in an increased accumulation of BBR in the axon terminals. As a consequence, anterograde mitochondrial transport was increased and axon-al retraction was suppressed.

We hypothesized that axon terminal-targeting LPs (BBR) could deliver BBR locally to axon terminals better compared with the use of naked BBR, which exhibits cationic properties. This could change the location of transient energy demand in the cell and affect mitochondrial dynamics and neurite length. We predicted that anterograde mitochondrial transport at the axon terminals would increase via the suppression of axonal retraction (Figure 1). It seemed logical that local drug delivery to neurons would be important in enhancing local pharmacological effects. Neuro2a cells, model neuroblastoma cells, and cultured primary neurons were used as representative targets in these experiments.

## 2. Materials and Methods

### 2.1. Materials

We obtained 1,2-dioleoyl-sn-glycero-3-phoshoetanolamine (DOPE) and methoxy polyethene glycol 2000 (DMG-PEG-2k) from the NOF Corporation (Tokyo, Japan). Sphingomyelin (SM) was obtained from Avanti Polar Lipids (Alabaster, AL, USA). Stearylated R8 (STR-R8) and axon-targeting peptides were obtained from KURABO Industries (Osaka, Japan). We obtained 1,1′-Dioctadecyl-3,3,3′,3′-Tetramethylindodicarbocyanine, 4-Chlorobenzenesulfonate (DiD), and a MitoTracker™ Green FM and B-27™ Plus Neuronal Culture System from Thermo Fisher Scientific Life Sciences (Waltham, MA, USA). Eagle’s Minimum Essential Medium (EMEM) was obtained from ATCC (Manassas, VA, USA). Fetal bovine serum (FBS) and Poly-L-ornithine were obtained from Sigma-Aldrich (St. Louis, MO, USA). All other chemicals used are commercially available, reagent-grade products. The flexiPERM was obtained from Sarstedt (Nümbrecht, Germany).

### 2.2. Preparation of the LPs

LPs were prepared via the lipid film hydration method. In a glass tube, a total of 900 nmol of lipids (DOPE/SM = 9/2, molar ratio) was dissolved in ethanol, and chloroform was added. A thin lipid film was formed by the evaporation of organic solvents for more than 2 h. DiD (0.5 mol% of total lipids) was served as the fluorescently labeled LPs. DiD, a carbocyanine dye, is retained on the particle membrane as the result of hydrophobic interactions during particle formation. Next, 10 mM of HEPES buffer (pH 7.4) containing berberine (600 µM) was added, which was followed by sonication for 1 min in a bath-type sonicator. Finally, to attach either R8 or axon terminal-targeting peptides to the surface of the LPs, a solution of each peptide (10 mol% of total lipids) was added to the resultant suspension, then the suspensions were left for 30 min at room temperature. The properties of the resultant LPs were determined using a Zetasizer Nano ZS (Malvern Panalytical, Malvern, UK). 

### 2.3. Cell Cultures

Neuro2a cells were obtained from ATCC (Manassas, VA, USA). Neuro2a cells were maintained in a complete medium that comprised of EMEM medium supplemented with 10% FBS, penicillin (100 U/mL), and streptomycin (100 µg/mL). The cells were cultured under an atmosphere of 5% CO_2_/air at 37 °C. One day prior to treatment of the samples, Neuro2a cells were seeded onto either plates or dishes for each experiment.

### 2.4. Culture of Primary Neurons

For primary cultures of neurons, P4 to P6 mice (Jcl:ICR mice) were obtained from Hokudo Co., Ltd., Sapporo, Japan. Cerebella were dissected following decapitation, and neurons were dissociated with trypsin and plated on glass that had been coated with poly-L-ornithine and attached to a silicon chamber (flexiPERM), as described previously [18,19]. Neurons were maintained in a B-27™ Plus Neuronal Culture System. At 1 day in vitro (DIV), cytosine β-D-arabinofuranoside (10 μM) was added to the culture, and the silicon chamber was removed to visualize the axonal extension. The cells were cultured under an atmosphere of 5% CO_2_/air at 37 °C. Four days prior to treatment of the samples, primary cultures of neurons were seeded onto either plates or dishes for each experiment.

### 2.5. Assessment of Cellular Uptake Using a Fluorescence Activated Cell Sorter (FACS)

Following washing with phosphate buffered saline without calcium chloride (PBS (-)), the media were replaced with the LPs (final lipid concentration: 0.36 µM) containing DMEM without serum, and were incubated for 30 min under an atmosphere of 5% CO_2_/air at 37 °C. Then, the media were replaced with a complete medium and incubated for 30 min under an atmosphere of 5% CO_2_;/air at 37 °C. The cells were washed twice with PBS (-) containing heparin (20 U/mL), and then collected with trypsin 0.25% EDTA. After centrifugation at 700× *g* and 4 °C for 3 min and removal of the supernatant, the collected cells were suspended in FACS buffer, which contained bovine serum albumin (5 mg/mL) and sodium azide (1 mg/mL) in PBS (-). After filtration through a nylon mesh, the cells were analyzed using a CytoFLEX Flow Cytometer (Backman Coulter Inc., Brea, CA, USA). The DiD was excited via 638 nm light, and the bandpass filter for the fluorescence detection was set to 660 nm. The values for the cellular uptake of each of the LPs were expressed as the MFI, the integrated fluorescence intensity, and the cell count.

### 2.6. Cell Imaging Using Confocal Laser Scanning Microscopy (CLSM)

Following washing with PBS (-), the media were replaced by the DiD (final lipid concentration: 0.36 µM) and DMEM without serum with incubation for 30 min under the same conditions as above. To stain the axonal mitochondria, neurons were treated with MitoTracker^TM^ Green FM (final concentration 0.5 µM) and incubated for 20 min under the same conditions as above. The media were replaced with a complete medium and washed twice with DMEM and 1 mL of fresh complete medium was added, observed using CLSM (Nikon A1; Nikon Company Ltd., Tokyo, Japan) equipped with an objective lens (Plan Flour ×40 objective lens/NA 1.3) and a dichroic mirror (405/473/559/635). The cells were excited with a 473 nm light and a 635 nm light from an LD laser. We mechanically analyzed the number of particles in the non-overlapping cell bodies and axons by particle analysis using ImageJ software (version 1.52a). The number of particles was calculated using the following equation.

Cell body uptake value = uptake cell number/all cell number × uptake particle volume − Axon terminal uptake value = uptake axon number/all axon number × uptake particle volume.

Time-lapse imaging was performed using CLSM at 8 s intervals for 5 min to evaluate the mitochondrial dynamics. In time-lapse imaging, all axons with mitochondria that exhibited small and rounded shapes, as well as no movement, were excluded from the analysis. Mitochondria were deemed to be stationary when the maximum change in position during observation was less than 5 µm on kymographs or intermittent images. We mechanically analyzed the mitochondrial dynamics using ImageJ software. Mitochondrial dynamics were calculated using the following equation.

Motility of mitochondria (%) = motility mitochondria/all mitochondria × 100

Anterograde of mitochondria (%) = anterograde mitochondria/motility mitochondria × 100.

### 2.7. Measurement of Neurite Length

Transfection was performed in the same manner as described in Section 2.6 and observed using CLSM (Nikon Ti; Nikon Company Ltd., Tokyo, Japan) equipped with an objective lens Plan Flour ×10 objective lens/NA 0.3) and a dichroic mirror (405/473/559/635). We mechanically analyzed the neurite length in non-overlapping axons using ImageJ software. The lengths of 8 or more axons were measured three times. The percentage of changes in axonal length was calculated using the following equation.

Changed axon length (%) = axon length after treatment with samples/axon length before treatment with samples × 100.

### 2.8. Statistical Analysis

All experiments were performed in triplicate on at least 3 independent days. One-way ANOVA was performed, which was followed by a Tukey’s test (R software (version 4.3.1)) for multiple comparisons. The levels of significance are denoted as follows: * *p* < 0.05, ** *p* < 0.01.

## 3. Results

### 3.1. Preparation of the Axon Terminal-Targeting LPs Using the Lipid Film Hydration Method

Axon terminal-targeting LPs were prepared using the thin film hydration method. The prepared LPs were composed of 1,2-dioleoyl-sn-glycero-3-phoshoetanolamine (DOPE) and Sphingomyelin (SM) (9:2 molar ratio). To accumulate LPs at the axon terminals, the LPs were modified with synapse-targeting peptides. Neurexin is one of the most studied and important factors in regulating synaptic function. Neurexin involves cell adhesion molecules that are abundantly expressed in axon terminals with three extracellular epidermal growth factor-like domains (EGF) and six laminin-neurexin sex-hormone-binding globulin domains (LNS). For the peptides targeting axon terminals, we used a Carbonic Anhydrase-related Protein (CAP), a secreted glycoprotein identified by the proteomic screening of neurexin [20], a Cysteine-Rich Region (CRR) derived from the N-terminal CRR of cerebellin [21], a Neurexide (NRX), a complex of neurexin and peptide [22], and Neurexin Binding Sites (NBS) derived from the NBS of neuroxophilin [23] for which an affinity for neurexin had already been reported (Table 1). The characteristics of the prepared LPs are summarized in Table 2. The axon terminals targeting LPs were either negatively or neutrally charged with a diameter of about 100~300 nm.

The lipid compositions of prepared LPs were DOPE and SM (9:2 molar ratio). Data represent the mean ± S.D. (*n* = 3).

### 3.2. Assessment of LP Uptake after Sample Treatment

**Figure 2:**
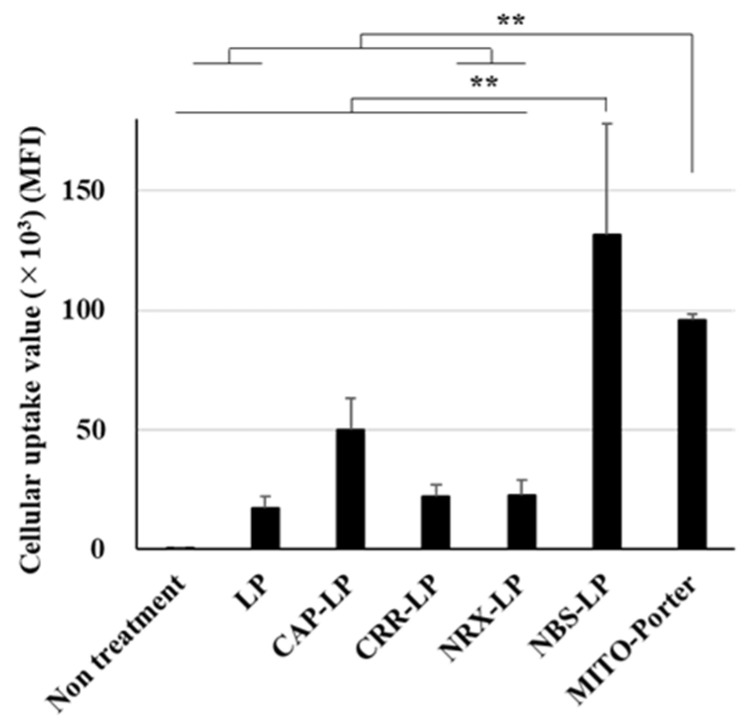
Assessment of the cellular uptake of LPs. Cellular uptake of LPs into Neuro2a cells was measured based on MFI using a FACS. LPs were labeled with DiD, a fluorescent dye, and the cells were analyzed after treatment of the LPs. Data are reported as the mean ± S.D. (*n* = 3). ** *p* < 0.01.

After treating Neuro2a cells with the LPs for 2 h, the cellular uptake was analyzed using FACS. The cellular uptake of the LPs is expressed as the mean fluorescent intensity (MFI). Untreated LPs were used as negative controls. LPs modified with NBS showed a high cellular uptake value (Figure 2). Moreover, the cellular uptake of the MITO-Porter was similar to that of the NBS-LP (Figure 2). This result raises the possibility that NBS-LP may be a useful carrier of LPs targeting neurons.

### 3.3. Observation of LPs in Subcellular Localization after the Treatment of Samples

**Figure 3:**
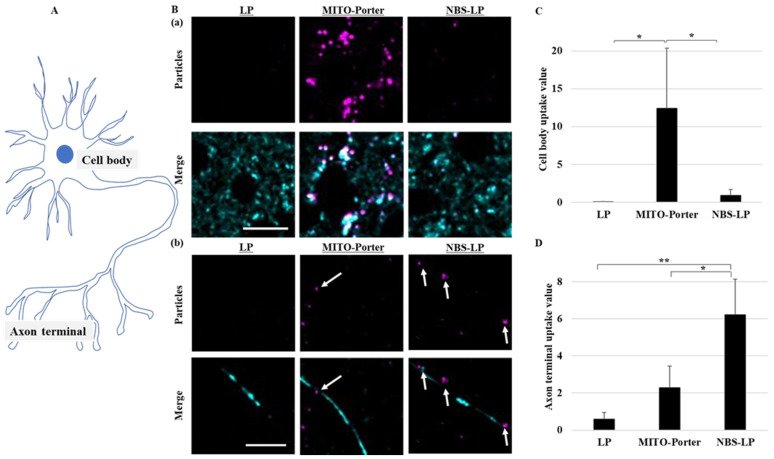
Assessment of the localization of LPs in the culture of primary neurons. Intracellular observation of nanoparticles was performed using CLSM. (**A**) Schematic diagram of neurons. (**B**) Representative images of a (**a**) cell body and (**b**) axon terminals of primary neurons labeled with MitoTracker Green (Cyan) and DiD (Purple). White arrows indicate the DiD-labeled nanoparticles. (**C**) Particle uptake values in the cell body. (**D**) Particle uptake values in the axon terminal. Scale bars indicate 20 µm. Data represent the mean ± S.D. (*n* = 3–4). * *p* < 0.05, ** *p* < 0.01.

The subcellular localization of LPs into the culture of primary neurons was evaluated using confocal laser scanning microscopy (CLSM). The culture of primary neurons was performed as previously described [18,19]. A distal axonal segment was defined as an axon terminal in this study (Figure 3A). After treating the culture of primary neurons with the LPs, neurons were treated with MitoTracker Green to stain axonal mitochondria. Many LPs were localized in the cell body after treatment with MITO-Porter (Figure 3B(a)). The amounts of LPs in the cell body were about 10-fold higher following treatment with MITO-Porter compared with those treated with either LP or NBS-LP (Figure 3C). In contrast, multiple LPs were localized in axon terminals after treatment with NBS-LP (Figure 3B(b)). The amounts of LPs in the axon terminals were about three-fold higher following treatment with NBS-LPs compared with those treated with MITO-Porter (Figure 3D). These results suggest that NBS-LPs could be used as a drug delivery carrier to axon terminals.

### 3.4. Evaluating the Dynamics of Axonal Mitochondria in the Samples following Treatment

**Figure 4:**
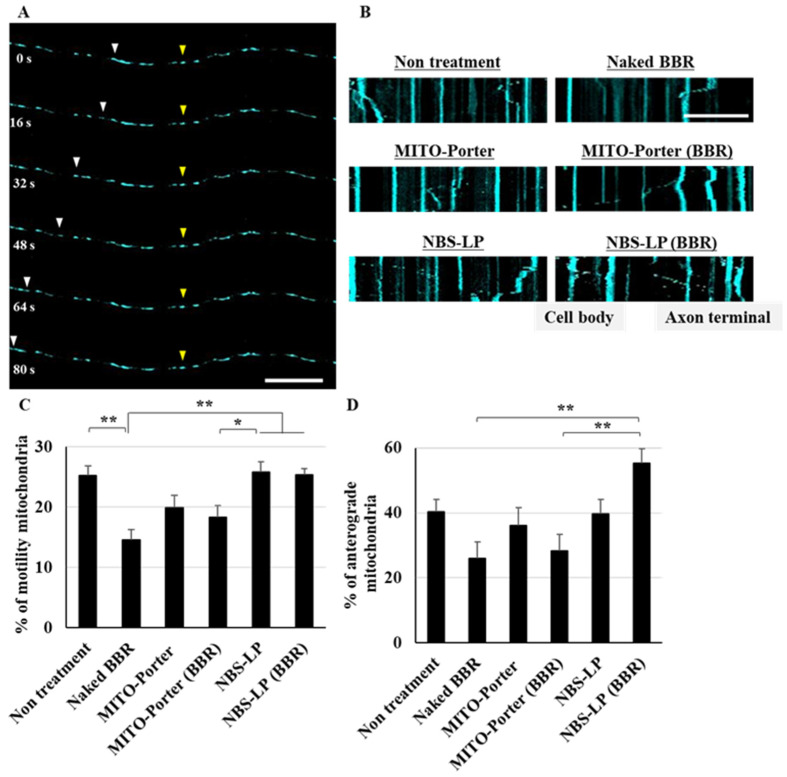
Evaluation of axonal mitochondrial transport. (**A**) Representative images of axonal mitochondria at the indicated time points. Yellow arrowheads indicate mitochondria that remained in the same position, whereas white arrowheads indicate mitochondria that changed position. (**B**) Representative kymographs of axonal mitochondria after treatment of these samples. (**C**) The percentage of motility mitochondria was quantified from the kymograph. (**D**) The percentage of anterograde mitochondria was quantified using the kymograph. Scale bars indicate 20 µm. Data are reported as the mean ± S.E. (*n* = 29–43). * *p* < 0.05, ** *p* < 0.01.

After treating the culture of primary neurons with the LPs, axonal mitochondrial transport was evaluated using time-lapse imaging via CLSM (Figure 4A). In a created kymograph using ImageJ software and time-lapse images (Figure 4B), mitochondria were defined as stationary if the change in position was less than 5 µm and motile when the change in position was more than 5 µm. Treatment with naked BBR or MITO-Porter (BBR) decreased axonal mitochondrial transport by about 40% compared with that of untreated samples (Figure 4C). In contrast, the rate of axonal mitochondrial transport after treatment with NBS-LP (BBR) was comparable to that of the untreated samples (Figure 4C). Moreover, we evaluated the rate of anterograde transport from total axonal mitochondrial transport. Treatment with NBS-LP (BBR) increased anterograde transport by about 40% compared with that of either naked BBR or MITO-Porter (BBR) (Figure 4D). These results suggest that local BBR delivery to axon terminals could locally regulate mitochondrial dynamics.

### 3.5. Evaluation of Axonal Outgrowth following the Treatment of Samples

**Figure 5:**
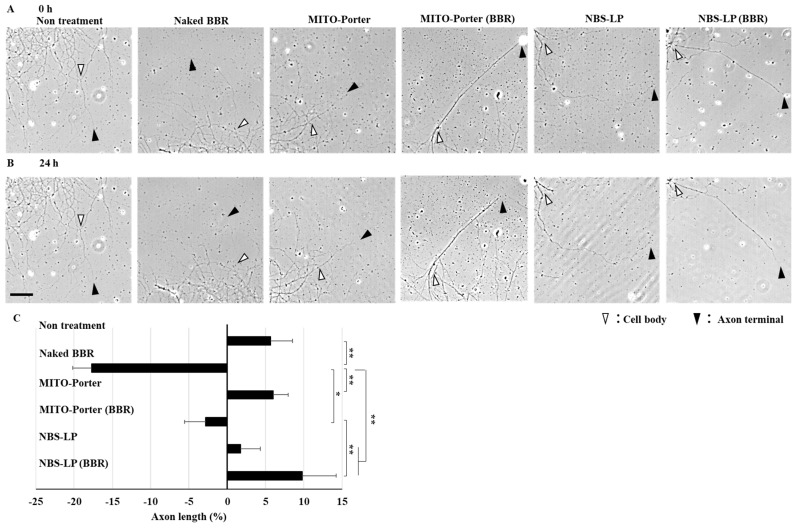
Evaluation of axon length. Representative images of the axons at the indicated time points (after treatment of these samples at 0 h (**A**) and 24 h (**B**)). White arrowheads indicate the cell body side, whereas open arrowheads indicate the axon terminal side. (**C**) The percentage of changed axon length was quantified using the bright field image. The scale bar indicates 40 µm. Data represent the mean ± S.E. (*n* = 25–44). * *p* < 0.05, ** *p* < 0.01.

The axons of the cultured primary neurons were observed just after or 24 h after treatment with LPs using CLSM. The axon lengths of the primary neurons were evaluated via the bright field images of two time points using ImageJ software (Figure 5A,B). Treatment with naked BBR decreased axon length by about 20% compared with that of untreated samples. Moreover, treatment with MITO-Porter (BBR) decreased axon length by about 10% compared with that of untreated samples. In contrast, treatment with NBS-LP (BBR) increased axon length by about 5% compared with that of untreated samples (Figure 5C). This result raises the possibility that the delivery of local BBR into axon terminals suppresses axonal retraction.

## 4. Discussion

This paper focuses on the question of whether nanoparticles could be used for local drug delivery to neurons. We postulated that NBS-LPs would accumulate near the axon terminals (Figure 3).

In the present study, MITO-Porter was highly localized in the cell body, but rarely localized in the axon terminals (Figure 3). MITO-Porter modified with R8 has a positively charged surface potential and increased the binding to negatively charged membrane surfaces such as cell membranes and mitochondrial outer membranes [16]. Previously, it was confirmed that cationic nanoparticles are highly localized in the cell bodies of neurons [24,25]. We decided that the binding of cationic nanoparticles decreases because there are many synapses at axon terminals where the membrane potential is more likely to fluctuate. These findings suggest that MITO-Porter is positively charged and thus localizes more in cell bodies compared with axon terminals.

Local changes in axon terminals are believed to play an important role in maintaining neuronal function, but the currently reported methods have limitations [3,4]. Therefore, we attempted to develop LPs for local drug delivery to axon terminals. Neurexin is ex-pressed in presynapses, which are abundant at axon terminals. As a result of modifying the surface of LPs with an axon terminal-targeting peptide (NBS) that has an affinity for neurexin, the number of particles at the axon terminals in the culture of primary neurons increased approximately three times compared with treatment using MITO-Porter (Figure 3D). These results suggest that both MITO-Porter and NBS-LP are able to locally deliver drugs to the cell body and axon terminals. However, we only observed the subcellular localization of particles; the localization of whole particles in the culture of neurons should be confirmed using microfluidic devices. Further studies will involve the direct evaluation of the physicochemical characterizations of the LNPs and the amount of BBR.

We focused on BBR influence in mitochondrial axonal transport because its acts on mitochondria and regulates intracellular energy homeostasis. Mitochondrial axonal transport contributes to overall cellular energy homeostasis by transporting mitochondria to sites of energy deprivation [1,2]. We predicted that transient changes in energy homeostasis caused by BBR-induced inhibition of the mitochondrial respiratory chain complex would lead to changes in mitochondrial axonal transport by changing the localization of BBR. Based on the results shown in Figure 3, we confirmed that MITO-Porter is non-selectively taken up into neurons and accumulates in the cell body, so we expected that BBR would be taken up into cells in a similar manner. Therefore, we evaluated mitochondrial axonal transport at axon terminals in response to local BBR delivery in the culture of primary neurons. Treatment with naked BBR decreased mitochondrial axonal transport at the axon terminals by about 40% (Figure 4C). This result could be attributed to increased energy demand in the cell body. Additionally, treatment with MITO-Porter (BBR), which also localizes to axon terminals, partially suppressed this decrease. This could be due to the high affinity of MITO-Porter (BBR) for mitochondria, which caused energy demand at axon terminals even at lower drug delivery doses compared with that of naked BBR. In contrast, treatment with NBS-LP (BBR) did not change mitochondrial axonal transport at the axon terminals (Figure 4C). These findings suggest that local drug delivery of BBR is important in altering mitochondrial axonal transport.

By activating AMPK, BBR may affect the activity of kinesin, which is the motor protein responsible for mitochondrial transport to axon terminals. We evaluated the mitochondrial dynamics in axons using the culture of primary neurons. The naked BBR and MITO porters (BBR) accumulated mostly in the cell body, suggesting that they could have decreased mitochondrial axonal transport to the axon terminals to satisfy energy demands in the cell body (Figure 4D). On the other hand, treatment with NBS-LP (BBR) accumulated mostly at the axon terminals, suggesting that it could have increased mitochondrial axonal transport to the axon terminals to meet energy demands (Figure 4D). Additionally, treatment with NBS-LP did not increase mitochondrial transport to the axon terminals. Our results suggest that local delivery of BBR may have changed the local mitochondrial dynamics.

Energy is required for structural changes such as neurite outgrowth and synapse formation. Mitochondrial axonal transport is altered in response to these changes [26]. Therefore, mitochondrial axonal transport changes due to local BBR delivery were expected to affect the axon length. We evaluated axon length using the culture of primary neurons because treatment with naked BBRs has been reported to modulate neurite length [10,11,12]. The use of naked BBR and MITO-Porter (BBR) induced axonal retraction compared with that in untreated samples (Figure 5C). However, MITO-Porter, which can deliver BBR to mitochondria more efficiently, was confirmed to partially suppress this effect. On the other hand, treatment with NBS-LP (BBR) increased anterograde mitochondrial axonal transport compared with that of treatment with both naked BBR and MITO-Porter (BBR). We surmised that this may have satisfied the energy demand at the axon terminals, thereby suppressing axonal retraction (Figure 5C). This is consistent with previous studies such as axonal regeneration [27], suggesting that local regulation of mitochondrial axonal transport plays an important role in changing neuronal function.

This study suggests that nanoparticles can be used for local drug delivery to neurons, where it would influence the pharmacological effects. In the future, studying the differences in pharmacological effects due to local drug delivery will be valuable as it could lead to the development of efficient treatments for neurodegenerative diseases.

## Figures and Tables

**Table 1:** Sequences of axon terminal-targeting compounds.

Compounds	Sequence
CAP10 [16]	MEIVWEVLFLLQANFIVCISAQQNSPKIHEGWWAYKEVVQGSFVPVPSFWGLVNSAWNLCSVGKRQSPVNIETSHMIFDPFLTPLRINTGGRKVSGTMYNTGRHVSLRLDKEHLVNISGGPMTYSHRLEEIRLHFGSEDSQGSEHLLNGQAFSGEVQLIHYNHELYTNVTEAAKSPNGLVVVSIFIKVSDSSNPFLNRMLNRDTITRITYKNDAYLLQGLNIEELYPETSSFITYDGSMTIPPCYETASWIIMNKPVYITRMQMHSLRLLSQNQPSQIFLSMSDNFRPVQPLNNRCIRTNINFSLQGKDCPNNRAQKLQYRVNEWLLK
CRR [17]	QNETEPIVLEGKCLVVCDSNPTGTALGI
Neurexide [18]	ARPSTRADRA
Neurexin binding site [19]	MQAACWYVLLLLQPTVYLVTCANLTNGGKSELLKSGSSKSTLKHIWTESSKDLSISRLLSQTFRGKENDTDLDLRYDTPEPYSEQDLWDWLRNSTDLQEPRPRAKRRPIVKTGKFKKMFGWGDFHSNIKTVKLNLLITGKIVDHGNGTFSVYFRHNSTGQGNVSVSLVPPTKIVEFDLAQQTVIDAKDSKSFNCRIEYEKVDKATKNTLNYDPSKTCYQEQTQSHVSWLCSKPFKVICIYISFYSTDYKLVQKVCPDYNYHSDTPYFPSG

**Table 2:** Physical properties of the LPs employed in this study.

LP Type	Diameters (nm)	Polydispersity Index (PdI)	ζ-Potential (mV)	Peptide Sequence
LP	102 ± 4.0	0.21 ± 0.03	−5.3 ± 1.4	-
CAP-LP	276 ± 29	0.57 ± 0.09	−1.9 ± 0.5	SLQGKDCPNN
CRR-LP	138 ± 14	0.22 ± 0.02	−3.7 ± 1.0	LEGKCLVVCD
NRX-LP	99 ± 3.0	0.25 ± 0.03	22 ± 0.6	ARPSTRADRA
NBS-LP	109 ± 4.0	0.24 ± 0.01	−2.4 ± 1.2	GDFHSNIKT
MITO-Porter	93 ± 1.0	0.28 ± 0.04	25 ± 1.9	RRRRRRRR

## Data Availability

Data will be made available on request.
